# The role of aggrecan in normal and osteoarthritic cartilage

**DOI:** 10.1186/s40634-014-0008-7

**Published:** 2014-07-16

**Authors:** Peter J Roughley, John S Mort

**Affiliations:** Shriners Hospital for Children and McGill University, Montreal, Quebec Canada

**Keywords:** Aggrecan, Link protein, Hyaluronan, Matrix metalloproteinase, Aggrecanase, Age changes, Articular cartilage, Osteoarthritis

## Abstract

Aggrecan is a large proteoglycan bearing numerous chondroitin sulfate and keratan sulfate chains that endow articular cartilage with its ability to withstand compressive loads. It is present in the extracellular matrix in the form of proteoglycan aggregates, in which many aggrecan molecules interact with hyaluronan and a link protein stabilizes each interaction. Aggrecan structure is not constant throughout life, but changes due to both synthetic and degradative events. Changes due to synthesis alter the structure of the chondroitin sulfate and keratan sulfate chains, whereas those due to degradation cause cleavage of all components of the aggregate. These latter changes can be viewed as being detrimental to cartilage function and are enhanced in osteoarthritic cartilage, resulting in aggrecan depletion and predisposing to cartilage erosion. Matrix metalloproteinases and aggrecanases play a major role in aggrecan degradation and their production is upregulated by mediators associated with joint inflammation and overloading. The presence of increased levels of aggrecan fragments in synovial fluid has been used as a marker of ongoing cartilage destruction in osteoarthritis. During the early stages of osteoarthritis it may be possible to retard the destructive process by enhancing the production of aggrecan and inhibiting its degradation. Aggrecan production also plays a central role in cartilage repair techniques involving stem cell or chondrocyte implantation into lesions. Thus aggrecan participates in both the demise and survival of articular cartilage.

## Background

This review describes the role of aggrecan in the function of articular cartilage and how this role is perturbed in the osteoarthritic (OA) joint. It explains the structure/function relationship between the various regions of the aggrecan molecules and how these are altered by synthetic and catabolic events that occur throughout life and in the diseased joint. It is clear that a view of cartilage function based on the contribution of one molecule will be somewhat biased, but it is also arguable that no molecule plays a more important role than aggrecan. Without it the tissue could not withstand the rigours of joint loading and its decreased abundance marks the onset of the tissue decline associated with the OA joint.

## Review

### Articular cartilage, aggrecan and OA

Articular cartilage is the white, smooth, lustrous connective tissue that covers the surfaces of bones where they meet in diarthrodial joints. It serves two major functions; to provide almost frictionless motion and to counteract the impact of the compressive forces experienced across the joint during use. The first of these functions is not usually associated with aggrecan, but relates to the unique collagen fiber organization at the articular surface and the presence of lubricin and hyaluronan (HA)/phospholipid complexes that aid in joint lubrication (Gleghorn *et al*. 2009; Wang *et al*. 2013a). However, aggrecan does play a role in fluid pressurization of the cartilage which supports the articular surface and so may facilitate its function (Dabiri and Li, 2013; Moore and Burris, 2014). The ability to resist compression is intimately associated with the high abundance of aggrecan throughout the extracellular matrix of articular cartilage beneath its superficial zone.

**Figure 1:**
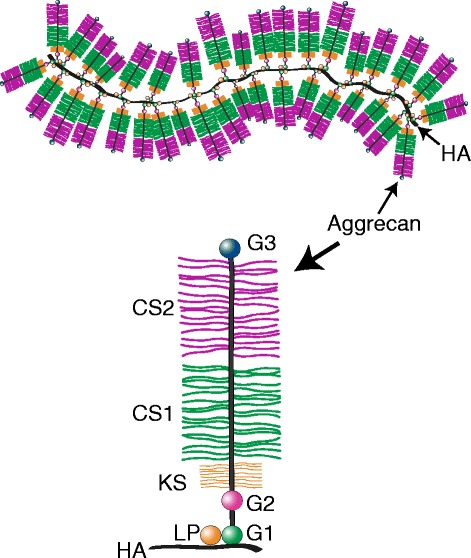
**Structure of proteoglycan aggregates.** The proteoglycan aggregate is depicted as a central hyaluronan (HA) filament with aggrecan and link proteins (LP) attached to it. The domains of the aggrecan core protein are indicated. G1, G2 and G3, globular regions; IGD, interglobular domain; KS, keratan sulfate-rich domain; CS1 and CS2, chondroitin sulfate-rich domains.

Aggrecan is a proteoglycan, and in common with all proteoglycans it possesses a core protein with covalently attached sulfated glycosaminoglycan (GAG) chains. Aggrecan does not exist in isolation within the extracellular matrix, but occurs in the form of proteoglycan aggregates (Hascall, 1988; Watanabe *et al*. 1998). Each aggregate is composed of a central filament of HA with multiple aggrecan molecules attached to it non-covalently via one terminus of their core proteins (Figure 1). The interaction between the aggrecan core protein and HA is stabilized by the presence of a link protein that interacts with both the aggrecan and HA. The GAG chains provide aggrecan with its high anionic charge whereas aggregation endows it with a large size. Both the charge and size properties are essential for normal aggrecan function and hence articular cartilage function.

The OA joint is characterized by catabolic processes that degrade both the HA backbone of the aggregate and the core protein of the aggrecan molecules, so impairing aggrecan function and predisposing the articular cartilage to erosion. Such degradation is associated with proteinases, hyaluronidases and free radicals.

### Structure of aggrecan

**Figure 2:**
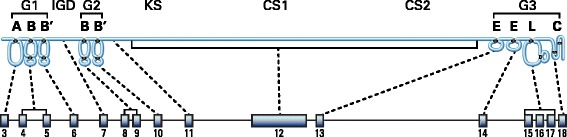
**Core protein domains of aggrecan.** Upper panel: The domains of the aggrecan core protein are depicted. G1, G2 and G3, globular regions; IGD, interglobular domain; KS, keratan sulfate-rich domain; CS1 and CS2, chondroitin sulfate-rich domains. The globular regions are divided into their disulfide-bonded domains. A, link protein-binding domain; B and B′, hyaluronan-binding domains for G1 and homology domains for G2; E, epidermal growth factor-like domains; L, lectin-like domain; C, complement regulatory protein-like domain. Lower panel: The coding exon arrangement of the human aggrecan gene is depicted and the regions of the core protein encoded by each exon is indicated.

The core protein of aggrecan consists of three disulfide-bonded globular regions (termed G1, G2 and G3) with intervening extended regions (Sandy *et al*. 1990) (Figure 2). The G1 region resides at the amino terminus of the core protein and is responsible for the interaction with HA. It is formed from three disulfide-bonded domains termed A, B and B′, with the B domains being responsible for the interaction with HA and the A domain being responsible for the interaction with link protein (Matsumoto *et al*. 2003; Watanabe *et al*. 1997). The G2 region is composed of two B-like domains, but does not have the ability to interact with HA and its functional role is at present unclear (Fosang and Hardingham, 1989). It is separated from the G1 region by the interglobular domain (IGD). The IGD is a prominent site for proteolysis, with many proteinases being able to cleave between the G1 and G2 regions (Fosang *et al*. 1992). The G1 and G2 regions and the IGD may be substituted with several N-linked and O-linked oligosaccharides or keratan sulfate (KS) (Barry *et al*. 1995).

The G2 and G3 regions are separated by a long GAG-attachment region that is subdivided into three domains (Doege *et al*. 1991). The KS-rich domain lies adjacent to the G2 region. It is composed of repeats of six amino acids, with each repeat possessing a proline-serine sequence. Each serine residue can potentially act as the attachment site for O-linked oligosaccharides that form the linkage region for KS. The KS-rich domain is followed by the chondroitin sulfate (CS)-rich domain, which is divided into two subdomains (CS1 and CS2) that differ in amino acid sequence. The CS1 domain is composed of repeats of nineteen amino acids, with each repeat possessing two serine-glycine sequences. Each serine residue in the repeats can potentially act as an attachment site for CS. The CS2 domain also possesses serine-glycine sequences that can act as the attachment site for CS. Because of their differing amino acid sequences, the CS1 and CS2 domains have differing susceptibilities to proteolysis. A typical aggrecan molecule may contain up to 100 CS chains and a lower number of KS chains. The sulfated nature of the CS and KS provide the aggrecan with its high anionic charge.

The CS2 domain is followed by the G3 region, which resides at the carboxy terminus of the core protein. The G3 region is composed of two epidermal growth factor (EGF)-like domains, one C-type lectin-like domain, and one complement regulatory protein (CRP)-like domain. The G3 region appears to be essential for normal trafficking of the aggrecan within the chondrocyte and for its secretion into the extracellular matrix (ECM) (Zheng *et al*. 1998). It is unclear whether each domain of the G3 region possesses a unique function within the ECM. The lectin-like domain does have the ability to interact with a variety of ECM proteins, such as tenascins and fibulins (Aspberg, 2012), and could potentially play a role in anchoring the aggrecan within the tissue. However, the G3 region is absent from many mature aggrecan molecules in the ECM due to its proteolytic cleavage (Dudhia *et al*. 1996a).

The domain structure of the aggrecan core protein is reflected in the exon organization of its gene (Valhmu *et al.*^1995^) (Figure 2). The human aggrecan gene consists of 19 exons. The G1 region is encoded by exons 3–6, with exon 3 encoding the A domain, exons 4 and 5 encoding the B domain and exon 6 encoding the B′ domain. The IGD is encoded by exon 7. The G2 region is encoded by exons 8–10, with exons 8 and 9 encoding the B domain and exon 10 encoding the B′ domain. The GAG attachment region is encoded by exons 11 and 12, with exon 11 encoding the first part of the KS-rich domain, and the large exon 12 encoding the remainder of the KS-rich domain plus the CS1 and CS2 domains. The hexapeptide repeats of the KS-rich region are encoded by exon 12. The G3 region is encoded by exons 13–19, with exons 13 and 14 each encoding an EGF-like domain, exons 15–17 encoding the lectin-like domain, and exon 18 encoding the CRP-like domain.

While the domain organization of aggrecan is conserved amongst different species, there are species variations in its core protein structure (Doege *et al*. 1987; Hering *et al*. 1997; Li *et al*. 1993; Walcz *et al*. 1994). Foremost amongst these are the variations in the number of repeats in both the KS-rich and CS domains. The number of KS-rich repeats varies from 4 in the rat to 23 in the bovine, with the human possessing 13 (Barry *et al*. 1994). In the CS1 domain there are 17 repeats in the rat, 23 in the mouse and 27 in the bovine. The human is unique in possessing length polymorphism in its CS1 domain, with the number of repeats varying from 13 to 33, though the majority of individuals have 26–28 repeats (Doege *et al*. 1997). In the human it has been shown that the KS-rich domain is devoid of CS and the CS1 domain is devoid of KS (Rodriguez *et al*. 2006). The human CS2 domain contains at least one KS chain adjacent to the G3 region. In the bovine this site is occupied by CS. The human also appears to be unique in possessing two exons encoding for EGF-like domains. In other species studied, genomic sequence variations prevent expression of the EGF1 domain and only the EGF2 domain is present (Fülöp *et al*. 1996). In the human, the G3 region has a variable structure due to alternative splicing of both EGF-like domains and the CRP-like domain (Grover and Roughley, 1993).

### Aggregation

The HA that forms the core of the proteoglycan aggregate is not unique to cartilage, but is ubiquitous in its presence (Fraser *et al*. 1997). It is formed at the plasma membrane of most cells by a hyaluronan synthase (Has) (Weigel and DeAngelis, 2007). Mammals possess 3 Has (Has1, Has2 and Has3) (Itano *et al*. 1999), with Has2 being the predominant form in cartilage (Recklies *et al*. 2001). The HA is synthesized in the cytosol and the growing chain is extruded directly into the extracellular environment (Hubbard *et al*. 2012), where it forms a coat around the chondrocytes (Knudson *et al*. 1999). It is not clear how HA is released from the cell surface or where interaction with aggrecan takes place. A typical HA chain may possess 10,000 disaccharide units, with each G1 domain interacting with a decasaccharide region (Hascall and Heinegård, 1974). Individual aggrecan molecules may be spaced about 50 disaccharides apart due to the size exclusion properties of their GAGs.

**Figure 3:**
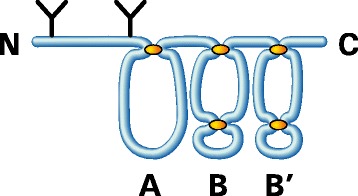
**Structure of link protein.** Link protein is depicted with three disulfide-bonded domains (A, B and B′) and two N-linked oligosaccharides (**Y**) in its amino terminal region. The amino terminus (N) and carboxy terminus (C) of the protein are indicated.

The link protein (LP) that stabilizes the proteoglycan aggregate has a domain structure analogous to that of the G1 region of aggrecan (Neame and Barry, 1993) (Figure 3). The A domain interacts with the A domain of the aggrecan G1 region and the B domains interact with HA. In the ECM, the link protein exists in three forms (LP1, LP2 and LP3) (Mort *et al*. 1985). LP1 has two N-linked oligosaccharides on residues 6 and 41, whereas LP2 has one N-linked oligosaccharide on residue 41. LP3 is formed by proteolytic cleavage between the two sites for N-linked oligosaccharide substitution. There is no evidence for any functional difference between the three LP forms. In addition to stabilizing the proteoglycan aggregates, the link protein also serves two additional functions. First, it forms a coat along the HA that helps protect the HA from degradation by hyaluronidases or free radicals (Rodriguez and Roughley, 2006). Second, it participates in the process of delayed aggregation (Oegema 1980). The G1 region of newly secreted aggrecan does not interact with HA, but acquires this ability following interaction with link protein in a process that appears to involve disulfide exchange within the G1 region (Melching and Roughley, 1990). Delayed aggregation may allow the aggrecan to escape retention by the HA coat at the cell surface of the chondrocyte and diffuse into the more remote ECM before aggregation occurs.

### Structure/function relationships of aggrecan

**Figure 4:**
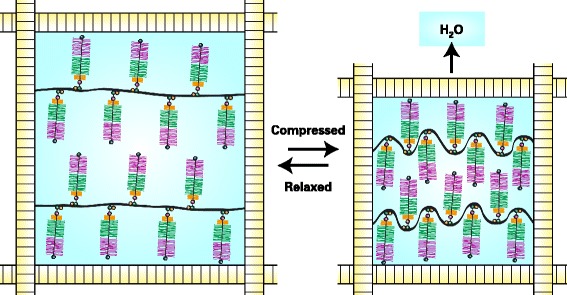
**The function of aggrecan in articular cartilage.** Proteoglycan aggregates are depicted as being entrapped by collagen fibrils. In the relaxed state the aggregates swell as the anionic chondroitin sulfate and keratan sulfate chains draw water into the tissue until an equilibrium is attained in which swelling is balance by tensile forces in the collagen fibrils. Under compression, water is displaced and the chondroitin sulfate and keratan sulfate chains are brought into closer proximity, so increasing their swelling potential and balancing the applied load. The increased swelling potential is dissipated upon removal of the load as the original equilibrium is restored.

When aggrecan finds itself in an aqueous environment its swells as the sulfated GAG chains become hydrated in an attempt to expand their molecular domain and increase their separation from one another. Within the ECM, such swelling is resisted by the collagen fibrils that form the framework of the cartilage. If sufficient aggrecan is present, an equilibrium will be attained with the swelling of the aggrecan being balanced by the tensile forces that it induces upon stretching of the collagen fibrils (Figure 4). For optimal cartilage function it is essential that aggrecan concentration be high enough so that such an equilibrium be attained. Upon compressing articular cartilage, the equilibrium is perturbed. Compression displaces water and as the size of the proteoglycan aggregates limits their free diffusion within the tissue, the aggrecan at the site of compression is bought into closer proximity. This increases the swelling potential of the aggrecan, and upon removal of the compression the aggrecan will re-swell and restore the original equilibrium. Thus normal articular cartilage function requires a high concentration of aggrecan, a high degree of aggrecan sulfation, and the ability to form large aggregates. Each of these properties is impaired in the OA joint.

The movement of water within the cartilage during cyclical compressive loading is thought to aid in the nutrition of the chondrocytes. As the cartilage is an avascular tissue, its nutrients must arise from the bathing synovial fluid by diffusion and its waste products must be expelled in a similar manner. The displacement of water upon compression aids in waste removal, and the return of water following removal of compression aids in nutrient supply. Obviously static loading cannot participate in such a mechanism. However, while dynamic loading may be beneficial to cartilage, its value will depend on its magnitude, frequency and duration. Loading at too high a magnitude may adversely influence the cells, resulting in cell death and ECM destruction. Cartilage overloading has long been thought to be linked to the onset of OA (Mitchell and Cruess, 1977).

### Variations in aggrecan structure due to synthesis

**Figure 5:**
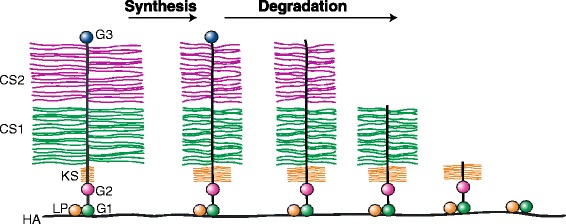
**Age-related changes in aggrecan structure.** Changes in aggrecan structure due to synthesis and degradation are depicted. Changes due to synthesis result in an increase in chondroitin sulfate length and a decrease in keratan sulfate length. Changes due to degradation result in truncation of the aggrecan core protein by removal of the G3 region, cleavage within the GAG-attachment region, and cleavage between the G1 and G2 regions.

The structure of aggrecan does not remain constant throughout life, but undergoes extensive changes due to variations in synthesis both within the chondrocytes and the ECM (Figure 5). The cellular changes due to synthesis take place during fetal life and postnatal growth and are essentially complete by 20 years of age. They affect mainly the length and sulfation pattern of the CS and KS chains on the aggrecan core protein (Brown *et al*. 1998; Plaas *et al*. 1997; Roughley *et al*. 1987; Roughley and White, 1980). With increasing age, the CS chains become shorter in length whilst the KS chains become longer. In the case of the KS chains it appears that sites occupied by O-linked oligosaccharides become occupied by KS, so increasing KS chain substitution (Santer *et al*. 1982). It is not clear if the pattern of CS chain substitution changes with age, or whether all potential CS attachment sites are always occupied. The divergent changes in CS and KS chain length may serve to maintain the charge of the aggrecan molecule. It has been suggested that the increase in KS reflects the avascular nature of mature articular cartilage, as KS does not require an oxidation step to generate a uronic acid for its constituent disaccharides (Balduini *et al*. 1992). The major change in sulfation pattern affects CS. In the human, the degree of sulfation increases throughout fetal life, and by birth 4 and 6-sulfation of the N-acetyl galactosamine residues occurs in equal abundance. Following birth, the sulfation pattern slowly changes to predominantly 6-sulfation. There is also a change in the sulfation pattern of KS, with increased sulfation of it galactose residues. It is not clear whether these changes in sulfation are of any significance in cartilage function.

**Figure 6:**
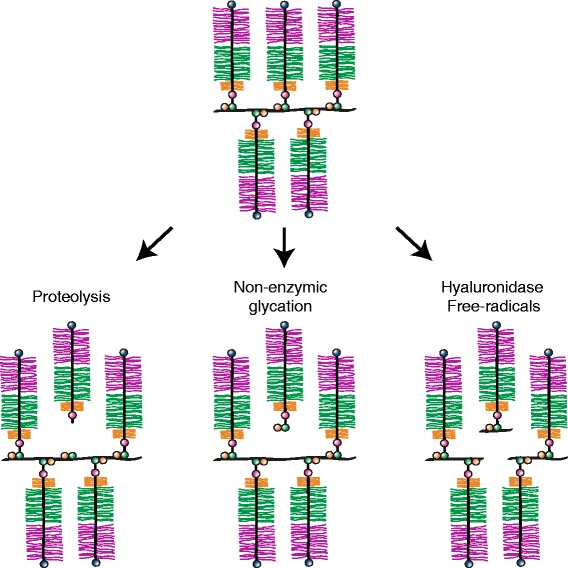
**Mechanisms of proteoglycan aggregate degradation.** A proteoglycan aggregate bearing five aggrecan molecules is depicted. Degradation is represented following proteolysis of the aggrecan core protein, non-enzymic glycation of the aggrecan core protein and link protein, and hyaluronidase or free radical cleavage of hyaluronan.

There is also one synthetic change in aggrecan structure that takes place in the ECM, involving non-enzymic glycation of the aggrecan core protein. This involves the interaction of a reducing sugar, such as glucose or ribose, with lysine residues in the core protein, and the ultimate formation of advanced glycation end-products (Cloos and Christgau, 2002), such as pentosidine, which accumulate with age (Verzijl *et al*. 2001). Such glycation can modify the lysine residues within the G1 region that facilitate the interaction with HA, and so prevent aggregation. More importantly, it can result in dissociation of the aggregates that are already formed (Figure 6). Thus glycation could adversely influence cartilage function.

### Variations in aggregate structure due degradation

The major changes in aggregate structure taking place in the ECM are due to degradation, and may affect all components of the aggregate (Figure 6). The protein/carbohydrate makeup of the proteoglycan aggregate renders it susceptible to multiple degradative mechanisms. Most importantly the protein component can be cleaved by a diverse series of proteolytic enzymes and the glycosaminoglycan component, in particular the HA, is susceptible to the action of glycosyl hydrolases. In addition, both the protein and polysaccharide constituents undergo modification by reactive oxygen species (ROS) which can lead to chain fragmentation.

The extended nature of the aggrecan core protein makes it a general target for degradation by proteolytic enzymes. The huge repertoire of proteases present in the body can be organized into six classes based on catalytic mechanism (Rawlings *et al*. 2012). *In vitro*, aggrecan is susceptible to cleavage by almost all known proteases and cleavage sites have been reported for members of four of these classes, serine, cysteine, aspartic and metalloproteases. In most cases the known selectivities of specific proteases are reflected in the novel N- and C-terminal sequences generated by their action. In the case of proteases known to be active in cartilage, correlation of such cleavage sites with the sequence termini determined in aggrecan fragments isolated from human articular cartilage and synovial fluid along with the detection of the cognate proteases *in situ* implicates the role of these proteases in cartilage degradation *in vivo* (Table 1). Such evidence points to a role for particular metallo-, cysteine and serine proteases in the degradation associated with aggrecan turnover in normal physiology and OA. Additional proteolytic cleavage sites have been identified by digestion of aggrecan *in vitro*, and the presence of the corresponding products *in vivo* has been inferred based on the molecular sizes of aggrecan fragments extracted from cartilage or isolated from synovial fluid (Struglics and Hansson, 2012).

**Table 1:** **Sequence termini on cleavage fragments of human aggrecan for which anti-neoepitope antibody reactivity, or direct sequencing, have demonstrated the presence of specific products** ***in vivo***

**Region**	**Sequence** ^**1**^	**Proteinase**	**Reference**
**IGD**	DIPEN^360^	MMP	(Flannery *et al*. 1992)
	^361^FFGVG	MMP	(Fosang *et al*. 1995)
	ITVQTV^375^	HtrA1	(Chamberland *et al*. 2009)
	ITEGE^392^	ADAMTS4/5	(Lark *et al*. 1997)
	^393^ARGSV	ADAMTS4/5	(Sandy *et al*. 1992)
**KS-rich**	VPGVA^709^	Calpain	(Maehara *et al*. 2007)
**CS1**	VGDLS^954^	Calpain	(Struglics *et al*. 2006)
**CS2**	ASELE^1564^	ADAMTS4/5	(Sandy and Verscharen, 2001)
	FKEEE^1733^	ADAMTS4/5	(Sandy and Verscharen, 2001)
	^1838^AGEGP	ADAMTS4/5	(Dufield *et al*. 2010)
	^1939^LGQRP	ADAMTS4/5	(Sandy and Verscharen, 2001)

^1^Aggrecan residue numbering is based on the human aggrecan sequence (Uniprot P16112, derived from the cDNA) which includes the signal peptide.

**Figure 7:**
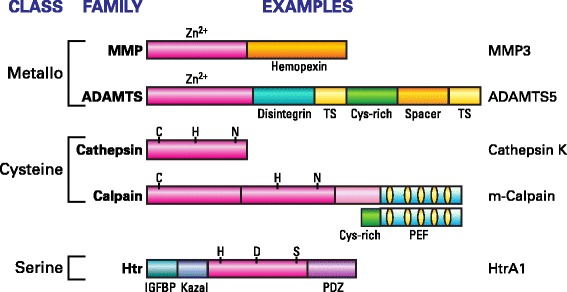
**Domain structure of proteases that participate in aggrecan core protein degradation.** The mature (active) forms of the enzymes are illustrated. The catalytic units are shown in magenta, with the catalytic amino acid residues (C, H, N, D,S) or co-factor (Zn^2+^) indicated. Ancillary domains, some of which are involved in substrate recognition, are also depicted. TS, thrombospondin-like; PEF, penta EF-hand (calcium binding domain); IGFBP, insulin-like growth factor binding protein-like; PDZ, protein/protein interaction domain. The functional form of calpain is a heterodimer of the large and small subunit shown, and that of HtrA1 is a homotrimer.

**Figure 8:**
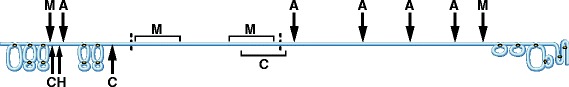
**Proteolytic cleavage sites within the aggrecan core protein.** The location of cleavage sites on the human aggrecan core protein determined *in vitro* by the action of proteases known to be active in cartilage *in vivo* are shown. A, ADAMTS4/5; C, calpain; H, HtrA1; M, MMPs.

Current evidence points to members of two families of metalloproteases, the MMP (matrix metalloproteinase) and ADAMTS (a disintegrin and metalloproteinase with thrombospondin motifs) families, as being the most important mediators of aggrecan degradation (Sztrolovics *et al.*^1997^). These are zinc ion dependent enzymes that are multidomain proteins where additional non-catalytic components serve to help select and anchor the substrate protein to the catalytic unit to facilitate proteolysis (Figure 7). MMPs 1, 8, and 13 are true collagenases, where their ancillary hemopexin domains are essential for the ability of these enzymes to degrade triple helical collagen. Other MMPs such as MMP3 (stromelysin) are effective general proteases and cleave at various sites along the aggrecan core protein, including the IGD (Figure 8) (Troeberg and Nagase, 2012). It has been proposed that MMPs are mainly involved in normal aggrecan turnover (Struglics and Hansson, 2012). Members of the ADAMTS family of metalloproteases (ADAMTS4 and 5) that were originally termed “aggrecanases” degrade aggrecan at a series of specific locations in the IGD and the CS2 domain. These cleavages have been shown to be particularly prevalent in the course of cartilage destruction in arthritis.

In addition to the metalloproteases, there is direct evidence for the involvement of specific cysteine and serine proteases in cartilage turnover. The presence of m-calpain in human cartilage has been reported for some time and it is clear that much of the fragmented aggrecan present in adult human cartilage, which consists of the G1-IGD-G2 regions, is the product of calpain action (Maehara *et al*. 2007). Calpain is a multidomain calcium dependent protease and would be active in the cartilage extracellular matrix. There is accumulating evidence that in addition to the action of MMP collagenases, the cysteine protease cathepsin K also plays a role in collagen breakdown in human cartilage. In contrast to the other proteases considered so far, cathepsin K consists of a single catalytic unit but forms a defined complex with chondroitin 4-sulfate which increases its stability and is essential for its ability to degrade triple helical collagen. Since cathepsin K is known to cleave aggrecan *in vitro* (Hou *et al*. 2003), it is likely to contribute to this process *in vivo*.

Recently direct analysis of human osteoarthritic cartilage extracts identified the interesting serine protease HtrA1 and aggrecan degradation products characteristic of its action in the IGD (Chamberland *et al*. 2009). Since elevated levels of this protease are seen in osteoarthritis (Grau *et al*. 2006) and given its ability to cleave many other extracellular matrix components (Eigenbrot *et al*. 2012), it may complement the well accepted role of the metalloproteases in disease progression.

While there is little evidence for degradation of CS and KS within the ECM, the size of the HA decreases with age (Holmes *et al*. 1988), presumably due to depolymerization by hyaluronidases or free radicals (Stern *et al*. 2007). A series of genes have been identified coding for glycosyl hydrolase-like proteins termed hyaluronidases (Csoka *et al*. 2001; Stern *et al*. 2007). However only two of these appear to be true hyaluronidases relevant to extracellular matrix degradation. While hyaluronidase-1 cleaves hyaluronan and chondroitin sulfate very effectively, it has a very low pH optimum as befits its normal lysosomal location. In contrast hyaluronidase-2 is active at neutral pH and at least a portion of it is located at the cell surface (Miller *et al*. 2006). Hyaluronidase-2 has been shown to cleave hyaluronan into relatively large fragments which are then taken up by the cell and degraded by hyaluronidase-1 and β-hexosaminidase (Gushulak *et al*. 2012).

Proteolysis causes extensive modification of the aggrecan core protein. This involves removal of the G3 region (Dudhia *et al*. 1996b), truncation within the GAG-attachment region and ultimately accumulation of G1 domains by cleavage within the IGD (Roughley *et al*. 1985) (Figure 5). In the case of link protein, proteolysis results in the accumulation of LP3 and a concomitant decrease in LP1 and LP2. *In vitro*, many proteinases are capable of forming LP3, but *in vivo* MMPs play a major role (Nguyen *et al*. 1991). Aggrecanases are not able to cleave link protein (Roughley *et al*. 2003). While cleavage of link protein may be of no functional consequence, cleavage of aggrecan and HA are detrimental to cartilage function as they reduce both the size and charge of the aggregates.

### Variations in aggrecan structure in OA

Articular cartilage in the OA joint is subjected to increased catabolism due to the presence of various cytokines that may arise from the chondrocytes themselves or by infiltration from the inflamed synovium (Goldring and Goldring, 2004). Foremost amongst these cytokines are interleukin 1 (IL1) and tumor necrosis factor α (TNFα), which not only stimulate the production of proteinases but also down-regulate aggrecan production. These inflammatory cytokines are associated with the production of aggrecanases and MMPs, which degrade the aggrecan core protein (Troeberg and Nagase, 2012). The combined effect of increased degradation coupled with decreased synthesis results in aggrecan loss from the ECM and impairment of articular cartilage function. Subsequently, collagenases (MMP1 and MMP13) degrade the collagen fibrils of the tissue, initiating tissue fibrillation and eventual erosion. Even if joint inflammation is resolved, it is likely that the depletion in aggrecan results in adverse consequences to loading, with the production of proteinases by the chondrocytes. The initiating event that causes aggrecan depletion may be either joint inflammation or joint overloading. Once damage is initiated, articular cartilage has little ability for endogenous repair and further damage appears to be an inevitable consequence.

While ineffective, articular cartilage does mount a limited repair response characterized by some cell proliferation and some new proteoglycan synthesis around the resulting cell clusters. Analysis of the aggrecan from OA cartilage shows a CS sulfation pattern more typical of juvenile cartilage than the mature adult (Caterson *et al*. 1990), suggesting that OA alters the phenotype of the resident mature chondrocytes or that it induces the formation of new immature chondrocytes. As articular cartilage is known to possess progenitor cells (Williams *et al*. 2010), these could be the source for cell proliferation and differentiation into immature chondrocytes.

### Retention and loss of aggrecan

Following proteolytic cleavage of the aggrecan core protein at any site, two fragments are generated. One fragment will possess a G1 region and remain bound to HA, whereas the other is no longer able to interact with HA. This latter non-aggregating fragment is free to diffuse within the ECM and is rapidly lost into the synovial fluid. In contrast, fragments that remain bound to HA can reside in the tissue for many years (Maroudas *et al*. 1998). It has been estimated that the G1 regions that form the end product of proteolytic truncation of the aggrecan core protein have a half-life of about 20 years. This can be viewed as an impediment to repair, as the G1 regions cannot participate in the load bearing function of the cartilage, yet they occupy space on the HA that could be used for binding newly synthesized aggrecan.

The aggrecan fragments that diffuse into the synovial fluid have been used as biomarkers in OA patients, with the premise that a higher concentration of fragments in the synovial fluid reflects enhanced degradation within the cartilage. This does assume that other factors, such as the rate of clearance from the synovial fluid or the secretion of newly synthesized aggrecan from the cartilage, are of lesser importance. In principle, the efficacy of drug therapy can be followed by monitoring the decline in fragment abundance. Fragment abundance can be monitored by a variety of techniques. The simplest techniques involve the quantification of sulfated GAG content by the dimethyl methylene blue (DMMB) assay (Farndale *et al*. 1986) and analysis of KS by immunoassay (Campion *et al*. 1991). An immune assay has also been described for a CS sulfation variant (846 epitope) that is characteristic of juvenile and OA cartilage (Poole *et al*. 1994). Finally, neoepitope immunoassays have been developed to specifically analyse for aggrecan degradation products resulting from either MMP or aggrecanase cleavage (Fosang *et al*. 2003; Germaschewski *et al*. 2014; Hughes *et al*. 1995).

### Repair of articular cartilage

While joint replacement with a prosthesis may be the preferred treatment for late stage OA where most of the articular cartilage has been eroded, biological repair is an attractive alternative for more focal early lesions. Current techniques tend to fall into two categories: those that are cartilage-based and those that are cell-based (Camp *et al*. 2014). The cartilage-based procedures include mosaicplasty, single osteochondral autografts and osteochondral allografts, where a mature articular cartilage is implanted into the lesion so that the composition of the repair tissue is equivalent to that which was eroded. The cell-based techniques include microfracture and autologous chondrocyte implantation (ACI), where the cells that will generate the repair cartilage are either bone marrow-derived mesenchymal stem cells or mature articular chondrocytes, respectively. When using stem cells, the structure and abundance of aggrecan and the composition of the cartilage ECM that is formed is immature and may not be ideal for withstanding the rigours of adult life. The use of mature chondrocytes may help resolve some of these problems, but the cartilage structure is still somewhat immature as age-dependent events, such as collagen cross-linking, that help reinforce the tissue are not fully established. A means of maturing the cell-derived cartilage would enhance its repair value. However, whichever technique is used, it is essential that the factor causing the degeneration be treated or eliminated if future degeneration of the repair tissue is to be avoided (Moran *et al*. 2014).

In the early stages of OA, prior to extensive collagen damage, it may be possible to retard or even reverse the degenerative process by the administration of agents that promote aggrecan synthesis and prevent its degradation. Development of specific small molecule inhibitors for MMP and ADAMTS family members has been an extremely active area for several decades. Although there have been setbacks (Clark and Parker, 2003), new highly specific compounds for the inhibition of ADAMTS5 are being produced (Deng *et al*. 2012) so there is still hope that products of this approach may reach the clinic. Nutriceuticals, such as glucosamine, have been touted as therapeutic agents, though their clinical efficacy remains to be proven (Salazar *et al.*^2014^). Some growth factors, such as transforming growth factor β (TGFβ) and bone morphogenetic protein 7 (BMP7) have the desired properties, but they are expensive to use and could have unwanted side effects. However, local delivery of single or a combination of growth factors, including insulin-like growth factor I (IGFI), fibroblast growth factor-2 (FGF2), TGFβ and BMPs, by way of gene therapy may overcome these deficits (Shi *et al*. 2012; Trippel *et al*. 2007). An economically attractive alternative to growth factors may be LinkN (McKenna *et al*. 1998), which represents the amino terminal 16 amino acids of link protein. It occurs naturally from the formation of LP3, but can be produced cheaply using a peptide synthesizer. It appears to function through the BMP receptor system (Wang *et al*. 2013b), but unlike BMPs does not stimulate osteogenesis (Antoniou *et al*. 2012). Only in the future will we know whether such agents live up to their current promise.

## Conclusions

Aggrecan plays an essential role in the function of articular cartilage and is a central participant in its destruction in the OA joint. Agents that prevent aggrecan degradation and restore its production may play a future role in the treatment of early stage OA.

## References

[CR1] Antoniou J, Wang HT, Alaseem AM, Haglund L, Roughley PJ, Mwale F (2012). The effect of Link N on differentiation of human bone marrow-derived mesenchymal stem cells. Arthritis Res Ther.

[CR2] Aspberg A (2012). The different roles of aggrecan interaction domains. J Histochem Cytochem.

[CR3] Balduini C, De LG, Passi A, Rindi S, Salvini R, Scott JE (1992). Effect of oxygen tension and lactate concentration on keratan sulphate and chondroitin sulphate biosynthesis in bovine cornea. Biochim Biophys Acta.

[CR4] Barry FP, Neame PJ, Sasse J, Pearson D (1994). Length variation in the keratan sulfate domain of mammalian aggrecan. Matrix Biol.

[CR5] Barry FP, Rosenberg LC, Gaw JU, Koob TJ, Neame PJ (1995). *N*- and *O*-linked keratan sulfate on the hyaluronan binding region of aggrecan from mature and immature bovine cartilage. J Biol Chem.

[CR6] Brown GM, Huckerby TN, Bayliss MT, Nieduszynski IA (1998). Human aggrecan keratan sulfate undergoes structural changes during adolescent development. J Biol Chem.

[CR7] Camp CL, Stuart MJ, Krych AJ (2014). Current concepts of articular cartilage restoration techniques in the knee. Sports Health.

[CR8] Campion GV, McCrae F, Schnitzer TJ, Lenz ME, Dieppe PA, Thonar EJ (1991). Levels of keratan sulfate in the serum and synovial fluid of patients with osteoarthritis of the knee. Arthritis Rheum.

[CR9] Caterson B, Mahmoodian F, Sorrell JM, Hardingham TE, Bayliss MT, Carney SL, Ratcliffe A, Muir H (1990). Modulation of native chondroitin sulfate structure in tissue development and in disease. J Cell Sci.

[CR10] Chamberland A, Wang E, Jones AR, Collins-Racie LA, LaVallie ER, Huang Y, Liu L, Morris EA, Flannery CR, Yang Z (2009). Identification of a novel HtrA1-susceptible cleavage site in human aggrecan. J Biol Chem.

[CR11] Clark IM, Parker AE (2003). Metalloproteinases: their role in arthritis and potential as therapeutic targets. Expert Opin Ther Targets.

[CR12] Cloos PA, Christgau S (2002). Non-enzymatic covalent modifications of proteins: mechanisms, physiological consequences and clinical applications. Matrix Biol.

[CR13] Csoka AB, Frost GI, Stern R (2001). The six hyaluronidase-like genes in the human and mouse genomes. Matrix Biol.

[CR14] Dabiri Y, Li LP (2013). Influences of the depth-dependent material inhomogeneity of articular cartilage on the fluid pressurization in the human knee. Med Eng Phys.

[CR15] Deng H, O'Keefe H, Davie CP, Lind KE, Acharya RA, Franklin GJ, Larkin J, Matico R, Neeb M, Thompson MM, Lohr T, Gross JW, Centrella PA, O'Donovan GK, Bedard KL, Van VK, Mataruse S, Skinner SR, Belyanskaya SL, Carpenter TY, Shearer TW, Clark MA, Cuozzo JW, Arico-Muendel CC, Morgan BA (2012). Discovery of highly potent and selective small molecule ADAMTS-5 inhibitors that inhibit human cartilage degradation via encoded library technology (ELT). J Med Chem.

[CR16] Doege K, Sasaki M, Horigan E, Hassell JR, Yamada Y (1987). Complete primary structure of the rat cartilage proteoglycan core protein deduced from cDNA clones. J Biol Chem.

[CR17] Doege KJ, Coulter SN, Meek LM, Maslen K, Wood JG (1997). A human-specific polymorphism in the coding region of the aggrecan gene: variable number of tandem repeats produce a range of core protein sizes in the general population. J Biol Chem.

[CR18] Doege KJ, Sasaki M, Kimura T, Yamada Y (1991). Complete coding sequence and deduced primary structure of the human cartilage large aggregating proteoglycan, aggrecan: human-specific repeats, and additional alternatively spliced forms. J Biol Chem.

[CR19] Dudhia J, Davidson CM, Wells TM, Hardingham TE, Bayliss MT (1996). Studies on the G3 domain of aggrecan from human cartilage. Ann NY Acad Sci.

[CR20] Dudhia J, Davidson CM, Wells TM, Vynios DH, Hardingham TE, Bayliss MT (1996). Age-related changes in the content of the C-terminal region of aggrecan in human articular cartilage. Biochem J.

[CR21] Dufield DR, Nemirovskiy OV, Jennings MG, Tortorella MD, Malfait AM, Mathews WR (2010). An immunoaffinity liquid chromatography-tandem mass spectrometry assay for detection of endogenous aggrecan fragments in biological fluids: use as a biomarker for aggrecanase activity and cartilage degradation. Anal Biochem.

[CR22] Eigenbrot C, Ultsch M, Lipari MT, Moran P, Lin SJ, Ganesan R, Quan C, Tom J, Sandoval W, van Lookeren CM, Kirchhofer D (2012). Structural and functional analysis of HtrA1 and its subdomains. Structure.

[CR23] Farndale RW, Buttle DJ, Barrett AJ (1986). Improved quantitation and discrimination of sulphated glycosaminoglycans by use of dimethylmethylene blue. Biochim Biophys Acta.

[CR24] Flannery CR, Lark MW, Sandy JD (1992). Identification of a stromelysin cleavage site within the interglobular domain of human aggrecan: evidence for proteolysis at this site in vivo in human articular cartilage. J Biol Chem.

[CR25] Fosang AJ, Hardingham TE (1989). Isolation of the N-terminal globular domains from cartilage proteoglycan: identification of G2 domain and its lack of interaction with hyaluronate and link protein. Biochem J.

[CR26] Fosang AJ, Last K, Gardiner P, Jackson DC, Brown L (1995). Development of a cleavage-site-specific monoclonal antibody for detecting metalloproteinase-derived aggrecan fragments: detection of fragments in human synovial fluid. Biochem J.

[CR27] Fosang AJ, Neame PJ, Last K, Hardingham TE, Murphy G, Hamilton JA (1992). The interglobular domain of cartilage aggrecan is cleaved by PUMP, gelatinases, and cathepsin B. J Biol Chem.

[CR28] Fosang AJ, Stanton H, Little CB, Atley LM (2003). Neoepitopes as biomarkers of cartilage catabolism. Inflamm Res.

[CR29] Fraser JR, Laurent TC, Laurent UB (1997). Hyaluronan: its nature, distribution, functions and turnover. J Intern Med.

[CR30] Fülöp C, Cs-Szabó G, Glant TT (1996). Species-specific alternative splicing of the epidermal growth factor-like domain 1 of cartilage aggrecan. Biochem J.

[CR31] Germaschewski FM, Matheny CJ, Larkin J, Liu F, Thomas LR, Saunders JS, Sully K, Whittall C, Boyle Y, Peters G, Graham NM (2014). Quantitation OF ARGS aggrecan fragments in synovial fluid, serum and urine from osteoarthritis patients. Osteoarthritis Cartilage.

[CR32] Gleghorn JP, Jones AR, Flannery CR, Bonassar LJ (2009). Boundary mode lubrication of articular cartilage by recombinant human lubricin. J Orthop Res.

[CR33] Goldring SR, Goldring MB (2004). The role of cytokines in cartilage matrix degeneration in osteoarthritis. Clin Orthop Relat Res.

[CR34] Grau S, Richards PJ, Kerr B, Hughes C, Caterson B, Williams AS, Junker U, Jones SA, Clausen T, Ehrmann M (2006). The role of human HtrA1 in arthritic disease. J Biol Chem.

[CR35] Grover J, Roughley PJ (1993). Versican gene expression in human articular cartilage and comparison of mRNA splicing variation with aggrecan. Biochem J.

[CR36] Gushulak L, Hemming R, Martin D, Seyrantepe V, Pshezhetsky A, Triggs-Raine B (2012). Hyaluronidase 1 and β-hexosaminidase have redundant functions in hyaluronan and chondroitin sulfate degradation. J Biol Chem.

[CR37] Hascall VC (1988). Proteoglycans: the chondroitin sulfate/keratan sulfate proteoglycans of cartilage. ISI Atlas Sci Biochem.

[CR38] Hascall VC, Heinegård D (1974). Aggregation of cartilage proteoglycans: II: oligosaccharide competitors of the proteoglycan-hyaluronic acid interaction. J Biol Chem.

[CR39] Hering TM, Kollar J, Huynh TD (1997). Complete coding sequence of bovine aggrecan: comparative structural analysis. Arch Biochem Biophys.

[CR40] Holmes MWA, Bayliss MT, Muir H (1988). Hyaluronic acid in human articular cartilage: age-related changes in content and size. Biochem J.

[CR41] Hou WS, Li Z, Büttner FH, Bartnik E, Brömme D (2003). Cleavage site specificity of cathepsin K toward cartilage proteoglycans and protease complex formation. Biol Chem.

[CR42] Hubbard C, McNamara JT, Azumaya C, Patel MS, Zimmer J (2012). The hyaluronan synthase catalyzes the synthesis and membrane translocation of hyaluronan. J Mol Biol.

[CR43] Hughes CE, Caterson B, Fosang AJ, Roughley PJ, Mort JS (1995). Monoclonal antibodies that specifically recognize neoepitope sequences generated by 'aggrecanase' and matrix metalloproteinase cleavage of aggrecan: application to catabolism *in situ* and *in vitro*. Biochem J.

[CR44] Itano N, Sawai T, Yoshida M, Lenas P, Yamada Y, Imagawa M, Shinomura T, Hamaguchi M, Yoshida Y, Ohnuki Y, Miyauchi S, Spicer AP, McDonald JA, Kimata K (1999). Three isoforms of mammalian hyaluronan synthases have distinct enzymatic properties. J Biol Chem.

[CR45] Knudson CB, Nofal GA, Pamintuan L, Aguiar DJ (1999). The chondrocyte pericellular matrix: a model for hyaluronan-mediated cell-matrix interactions. Biochem Soc Trans.

[CR46] Lark MW, Bayne EK, Flanagan J, Harper CF, Hoerrner LA, Hutchinson NI, Singer II, Donatelli SA, Weidner JR, Williams HR, Mumford RA, Lohmander LS (1997). Aggrecan degradation in human cartilage: evidence for both matrix metalloproteinase and aggrecanase activity in normal, osteoarthritic, and rheumatoid joints. J Clin Invest.

[CR47] Li H, Schwartz NB, Vertel BM (1993). cDNA cloning of chick cartilage chondroitin sulfate (aggrecan) core protein and identification of a stop codon in the aggrecan gene associated with the chondrodystrophy, nanomelia. J Biol Chem.

[CR48] Maehara H, Suzuki K, Sasaki T, Oshita H, Wada E, Inoue T, Shimizu K (2007). G1-G2 aggrecan product that can be generated by m-calpain on truncation at Ala^709^-Ala^710^ is present abundantly in human articular cartilage. J Biochem (Tokyo).

[CR49] Maroudas A, Bayliss MT, Uchitel-Kaushansky N, Schneiderman R, Gilav E (1998). Aggrecan turnover in human articular cartilage: use of aspartic acid racemization as a marker of molecular age. Arch Biochem Biophys.

[CR50] Matsumoto K, Shionyu M, Go M, Shimizu K, Shinomura T, Kimata K, Watanabe H (2003). Distinct interaction of versican/PG-M with hyaluronan and link protein. J Biol Chem.

[CR51] McKenna LA, Liu H, Sansom PA, Dean MF (1998). An N-terminal peptide from link protein stimulates proteoglycan biosynthesis in human articular cartilage in vitro. Arthritis Rheum.

[CR52] Melching LI, Roughley PJ (1990). Studies on the interaction of newly secreted proteoglycan subunits with hyaluronate in human articular cartilage. Biochim Biophys Acta.

[CR53] Miller AD, Vigdorovich V, Strong RK, Fernandes RJ, Lerman MI (2006). Hyal2, where are you?. Osteoarthritis Cartilage.

[CR54] Mitchell NS, Cruess RL (1977). Classification of degenerative arthritis. Can Med Assoc J.

[CR55] Moore AC, Burris DL (2014). An analytical model to predict interstitial lubrication of cartilage in migrating contact areas. J Biomech.

[CR56] Moran CJ, Pascual-Garrido C, Chubinskaya S, Potter HG, Warren RF, Cole BJ, Rodeo SA (2014). Restoration of articular cartilage. J Bone Joint Surg Am.

[CR57] Mort JS, Caterson B, Poole AR, Roughley PJ (1985). The origin of human cartilage proteoglycan link-protein heterogeneity and fragmentation during aging. Biochem J.

[CR58] Neame PJ, Barry FP (1993). The link proteins. Experimentia.

[CR59] Nguyen Q, Liu J, Roughley PJ, Mort JS (1991). Link protein as a monitor *in situ* of endogenous proteolysis in human articular cartilage. Biochem J.

[CR60] Oegema TR (1980). Delayed formation of proteoglycan aggregate structures in human articular cartilage disease states. Nature.

[CR61] Plaas AHK, Wong-Palms S, Roughley PJ, Midura RJ, Hascall VC (1997). Chemical and immunological assay of the nonreducing terminal residues of chondroitin sulfate from human aggrecan. J Biol Chem.

[CR62] Poole AR, Ionescu M, Swan A, Dieppe PA (1994). Changes in cartilage metabolism in arthritis are reflected by altered serum and synovial fluid levels of the cartilage proteoglycan aggrecan: implications for pathogenesis. J Clin Invest.

[CR63] Rawlings ND, Barrett AJ, Bateman A (2012). MEROPS: the database of proteolytic enzymes, their substrates and inhibitors. Nucleic Acids Res.

[CR64] Recklies AD, White C, Melching LI, Roughley PJ (2001). Differential regulation and expression of hyaluronan synthases in human articular chondrocytes, synovial cells and osteosarcoma cells. Biochem J.

[CR65] Rodriguez E, Roughley P (2006). Link protein can retard the degradation of hyaluronan in proteoglycan aggregates. Osteoarthritis Cartilage.

[CR66] Rodriguez E, Roland SK, Plaas A, Roughley PJ (2006). The glycosaminoglycan attachment regions of human aggrecan. J Biol Chem.

[CR67] Roughley PJ, Barnett J, Zuo F, Mort JS (2003). Variations in aggrecan structure modulate its susceptibility to aggrecanases. Biochem J.

[CR68] Roughley PJ, White RJ (1980). Age-related changes in the structure of the proteoglycan subunits from human articular cartilage. J Biol Chem.

[CR69] Roughley PJ, White RJ, Glant TT (1987). The structure and abundance of cartilage proteoglycan during early development of the human fetus. Ped Res.

[CR70] Roughley PJ, White RJ, Poole AR (1985). Identification of a hyaluronic acid-binding protein that interferes with the preparation of high-buoyant-density proteoglycan aggregates from adult human articular cartilage. Biochem J.

[CR71] Salazar J, Bello L, Chavez M, Anez R, Rojas J, Bermudez V (2014). Glucosamine for osteoarthritis: biological effects, clinical efficacy, and safety on glucose metabolism. Arthritis.

[CR72] Sandy JD, Flannery CR, Boynton RE, Neame PJ (1990). Isolation and characterization of disulfide-bonded peptides from the three globular domains of aggregating cartilage proteoglycan. J Biol Chem.

[CR73] Sandy JD, Flannery CR, Neame PJ, Lohmander LS (1992). The structure of aggrecan fragments in human synovial fluid. Evidence for the involvement in osteoarthritis of a novel proteinase which cleaves the Glu 373-Ala 374 bond of the interglobular domain. J Clin Invest.

[CR74] Sandy JD, Verscharen C (2001). Analysis of aggrecan in human knee cartilage and synovial fluid indicates that aggrecanase (ADAMTS) activity is responsible for the catabolic turnover and loss of whole aggrecan whereas other protease activity is required for C-terminal processing in vivo. Biochem J.

[CR75] Santer V, White RJ, Roughley PJ (1982). *O*-linked oligosaccharides of human articular cartilage proteoglycan. Biochim Biophys Acta.

[CR76] Shi S, Mercer S, Eckert GJ, Trippel SB (2012). Regulation of articular chondrocyte aggrecan and collagen gene expression by multiple growth factor gene transfer. J Orthop Res.

[CR77] Stern R, Kogan G, Jedrzejas MJ, Soltes L (2007). The many ways to cleave hyaluronan. Biotechnol Adv.

[CR78] Struglics A, Hansson M (2012). MMP proteolysis of the human extracellular matrix protein aggrecan is mainly a process of normal turnover. Biochem J.

[CR79] Struglics A, Larsson S, Pratta MA, Kumar S, Lark MW, Lohmander LS (2006). Human osteoarthritis synovial fluid and joint cartilage contain both aggrecanase- and matrix metalloproteinase-generated aggrecan fragments. Osteoarthritis Cartilage.

[CR80] Sztrolovics R, Alini M, Roughley PJ, Mort JS (1997). Aggrecan degradation in human intervertebral disc and articular cartilage. Biochem J.

[CR81] Trippel S, Cucchiarini M, Madry H, Shi S, Wang C (2007). Gene therapy for articular cartilage repair. Proc Inst Mech Eng H.

[CR82] Troeberg L, Nagase H (2012). Proteases involved in cartilage matrix degradation in osteoarthritis. Biochim Biophys Acta.

[CR83] Valhmu WB, Palmer GD, Rivers PA, Ebera S, Cheng J-F, Fischer S, Ratcliffe A (1995). Structure of the human aggrecan gene: exon-intron organization and association with the protein domains. Biochem J.

[CR84] Verzijl N, Degroot J, Bank RA, Bayliss MT, Bijlsma JW, Lafeber FP, Maroudas A, TeKoppele JM (2001). Age-related accumulation of the advanced glycation endproduct pentosidine in human articular cartilage aggrecan: the use of pentosidine levels as a quantitative measure of protein turnover. Matrix Biol.

[CR85] Walcz E, Deak F, Erhardt P, Coulter SN, Fülöp C, Horvath P, Doege KJ, Glant TT (1994). Complete coding sequence, deduced primary structure, chromosomal localization, and structural analysis of murine aggrecan. Genomics.

[CR86] Wang M, Liu C, Thormann E, Dèdinaitè A (2013). Hyaluronan and phospholipid association in biolubrication. Biomacromolecules.

[CR87] Wang Z, Weitzmann MN, Sangadala S, Hutton WC, Yoon ST (2013). Link protein N-terminal peptide binds to bone morphogenetic protein (BMP) type II receptor and drives matrix protein expression in rabbit intervertebral disc cells. J Biol Chem.

[CR88] Watanabe H, Yamada Y, Kimata K (1998). Roles of aggrecan, a large chondroitin sulfate proteoglycan, in cartilage structure and function. J Biochem (Tokyo).

[CR89] Watanabe H, Cheung SC, Itano N, Kimata K, Yamada Y (1997). Identification of hyaluronan-binding domains of aggrecan. J Biol Chem.

[CR90] Weigel PH, De Angelis PL (2007). Hyaluronan synthases: a decade-plus of novel glycosyltransferases. J Biol Chem.

[CR91] Williams R, Khan IM, Richardson K, Nelson L, McCarthy HE, Analbelsi T, Singhrao SK, Dowthwaite GP, Jones RE, Baird DM, Lewis H, Roberts S, Shaw HM, Dudhia J, Fairclough J, Briggs T, Archer CW (2010). Identification and clonal characterisation of a progenitor cell sub-population in normal human articular cartilage. PLoS One.

[CR92] Zheng J, Luo W, Tanzer ML (1998). Aggrecan synthesis and secretion: a paradigm for molecular and cellular coordination of multiglobular protein folding and intracellular trafficking. J Biol Chem.

